# A feasibility study of functional preservation in craniospinal irradiation with photon for pediatric medulloblastoma

**DOI:** 10.1002/acm2.70474

**Published:** 2026-01-27

**Authors:** Keqiang Wang, Jie Chen, Jianbo Jian, Peng Wang, Hongyang Zhang, Wenxue Zhang

**Affiliations:** ^1^ Department of Radiation Oncology Tianjin Medical University General Hospital Tianjin China

**Keywords:** craniospinal irradiation, functional preservation, NTCP, pediatric medulloblastoma, plan complexity, plan robustness

## Abstract

**Background:**

Craniospinal irradiation (CSI) is essential for treating pediatric medulloblastoma (MB) but causes significant long‐term toxicities. Existing dose‐reduction or partial‐sparing strategies improve neurocognitive outcomes but may compromise survival or fail to address other late effects.

**Methods:**

A new functional preservation CSI (FP‐CSI) technique was developed to spare the hippocampus, hypothalamic‐pituitary axis (HPA), cochlea, and scalp while ensuring homogeneous vertebral coverage. Eight pediatric patients with average‐risk MB were retrospectively planned with volumetric modulated arc therapy (VMAT) using both FP‐CSI and standard CSI (S‐CSI). Dosimetric parameters for the planning target volume (PTV) and organs at risk (OARs), radiobiological effects, plan robustness, plan complexity, and plan quality assurance (QA) were compared.

**Results:**

FP‐CSI significantly reduced mean doses to the hippocampus (12.4 vs. 23.9 Gy), hypothalamus (14.7 vs. 23.9 Gy), and pituitary gland (15.4 vs. 24.1 Gy, all *p* < 0.01). Vertebral dose gradients were halved (4.7 vs. 8.7 Gy). Moderate dose reductions were also achieved for the cochlea and scalp. Compared with S‐CSI, FP‐CSI exhibited slightly inferior PTV homogeneity (HI: 0.16 vs. 0.07) and conformity (CI: 0.88 vs. 0.93), but coverage remained clinically acceptable. Normal tissue complication probability (NTCP) modeling showed pronounced decreases in predicted neurocognitive and endocrine toxicity risks, with probability of neurocognitive impairment reduced from 84.5% to 24.9% and probability of endocrine dysfunction from 44.7% to 27.3%. FP‐CSI increased modulation complexity and produced slightly lower gamma passing rates for cranial beams, while spinal beam deliverability remained similar to S‐CSI. Robustness analysis indicated greater sensitivity of FP‐CSI to setup and rotational errors. Nevertheless, 3D dose reconstruction confirmed accurate delivery, with volumetric dose deviations generally below 1 Gy.

**Conclusion:**

FP‐CSI effectively spares critical functional structures while maintaining clinically acceptable target coverage, and offers a promising strategy to reduce long‐term radiotherapy‐induced toxicities in pediatric MB.

## 1 INTRODUCTION

Medulloblastoma (MB) is the most common malignant brain tumor in children and remains a leading cause of morbidity and mortality in this population. Arising predominantly in the cerebellum, MB frequently disseminates through the cerebrospinal fluid (CSF). Standard therapy consists of a multimodal regimen including surgical resection, craniospinal irradiation (CSI), and chemotherapy, with an additional boost to the tumor bed.^1^ Although advances in treatment have substantially improved survival, long‐term survivors often suffer from debilitating late effects, such as cognitive decline, endocrine dysfunction, scoliosis, hearing loss, and permanent alopecia, all of which severely impact quality of life.^2^, ^3^, ^4^, ^5^, ^6^, ^7^

Reducing the long‐term toxicity of CSI has therefore become a major focus of investigation. In standard CSI (S‐CSI) for average‐risk MB, the clinical target volume (CTV) encompasses the entire arachnoid space, with a prescribed dose of 23.4 Gy. Strategies to mitigate adverse effects include lowering the CSI dose and minimizing exposure to critical organs at risk (OARs). The Children's Oncology Group ACNS 0331 trial evaluated dose reduction in children aged 3–7 years with average‐risk MB, comparing 18 Gy to the conventional 23.4 Gy. While the reduced dose improved neurocognitive outcomes, it was associated with inferior event‐free survival, underscoring the urgent need for approaches that preserve efficacy while sparing healthy tissues.^8^ One promising method is hippocampal avoidance (HA) radiotherapy, designed to mitigate cognitive decline by sparing the hippocampus.^9^ Additionally, advanced delivery techniques, such as volumetric modulated arc therapy (VMAT), helical tomotherapy (HT), and intensity‐modulated proton therapy (IMPT), have demonstrated the capability to reduce radiation exposure to the hippocampus and hypothalamic‐pituitary axis (HPA) below 18 Gy, while still providing acceptable CSI target coverage, with D_95%_ exceeding 98% of the standard 23.4 Gy.^10^, ^11^

Beyond neurocognitive and endocrine sequelae, CSI contributes to significant physical late effects. Paulino et al.^4^ reported a 34.6% cumulative incidence of scoliosis at 15 years following photon CSI in pediatric patients. High‐dose radiation has also been identified as a major risk factor for permanent alopecia.^7^ These findings highlight the importance of comprehensive strategies that address multiple late toxicities. However, no existing approach has simultaneously demonstrated the ability to preserve hippocampal and HPA function, reduce risks of scoliosis, hearing loss, and alopecia, and still ensure adequate CSI coverage.

To address this gap, we propose a new strategy termed functional preservation CSI (FP‐CSI). This technique integrates advanced planning approaches to selectively spare critical OARs, including the hippocampus, HPA, scalp, and cochlea, while ensuring homogeneous vertebral coverage. The objectives of this study were threefold: (1) to establish delineation and planning parameters for FP‐CSI using VMAT, (2) to assess its dosimetric performance in sparing OARs while maintaining target volume coverage and vertebral dose homogeneity, and (3) to compare FP‐CSI with conventional S‐CSI in terms of dosimetry, radiobiological effects, plan robustness, plan complexity, and plan quality assurance (QA).

## 2 MATERIALS AND METHODS

### 2.1 Patient selection and CT simulation

This study included eight pediatric patients (aged 3–17 years) diagnosed with average‐risk MB, who underwent CSI in our hospital between 2018 and 2023. All patients were immobilized in the supine position using thermoplastic head‐and‐neck masks and knee supports, with their arms positioned alongside the thighs. CT simulation was performed using a CT simulator (Brilliance CT Big Bore, Philips Healthcare, Cleveland, OH) with a 3‐mm slice thickness covering the entire craniospinal axis. Brain MRI T1‐weighted and lumbar spine MRI T2‐weighted sequences were semi‐automatically fused with the CT simulation images using MIM software (MIM Maestro, version 7.3.4, Cleveland, OH) to enhance target delineation accuracy.

### 2.2 Target delineation

#### 2.2.1 Target in S‐CSI

**FIGURE 1:**
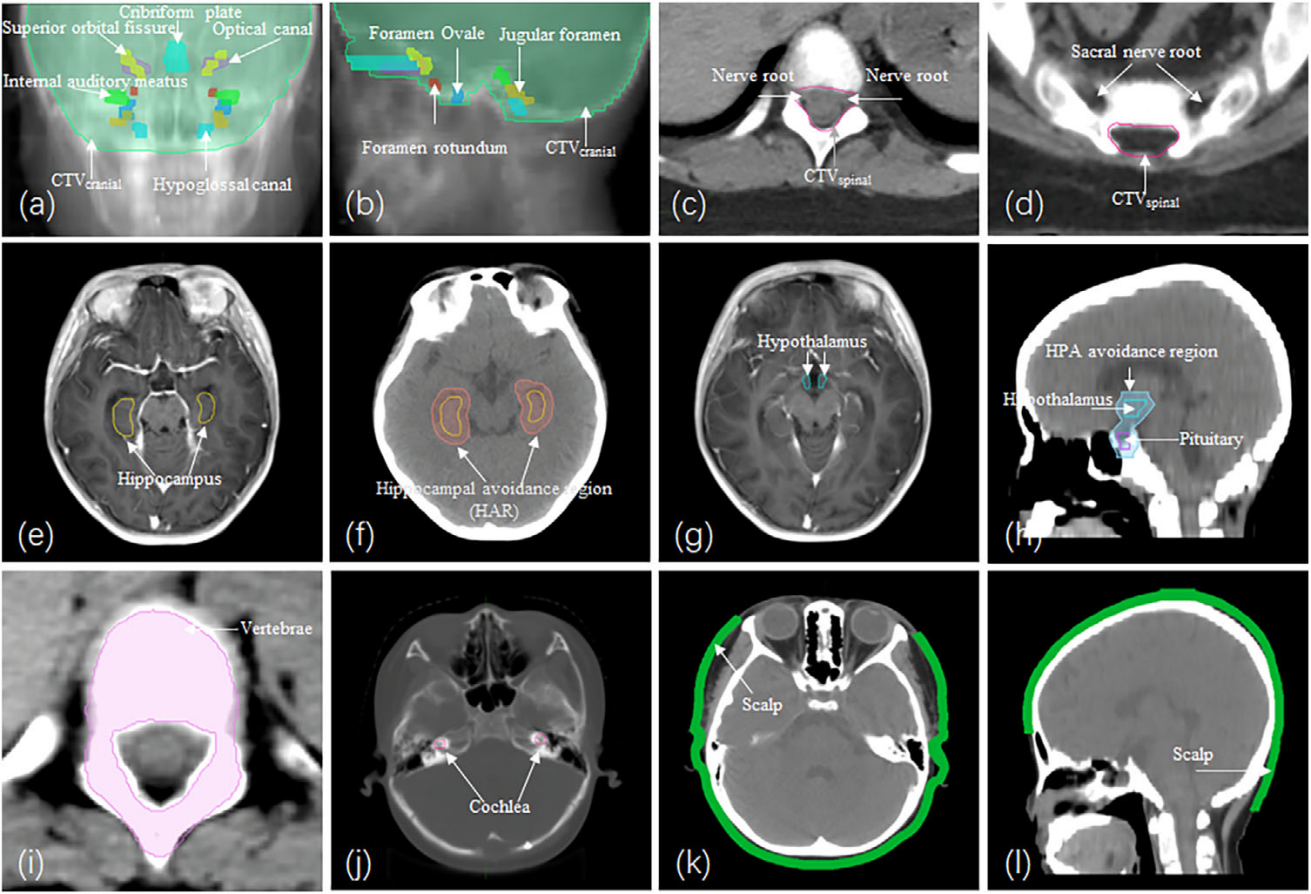
Illustrations of CTV and OARs delineation for CSI. (a, b) Coronal (a) and sagittal (b) projection of the cranial CTV, including skull base foramina. (c) Spinal CTV encompassing the entire arachnoid space with nerve roots. (d) Spinal CTV excluding sacral nerve roots. (e) Hippocampal delineation on MRI. (f) Hippocampus and hippocampal avoidance region (HAR) displayed on a CT image. (g) Hypothalamic volume delineation on MRI. (h) Hypothalamic‐pituitary axis (HPA) avoidance region on a CT image. (i) Vertebral volume delineation on a CT image. (j) Cochlear delineation on a CT image. (k, l) Coronal (k) and sagittal (l) views of the scalp delineation on CT images. CTV, clinical target volume; OARs, organs at risk; CSI, craniospinal irradiation.

The clinical target volume (CTV) encompassed the entire arachnoid space due to the high risk of CSF‐mediated disease dissemination. The cranial CTV (CTV_cranial_) was delineated according to the European Society for Pediatric Oncology (SIOPE) consensus guidelines, incorporating key CSF‐containing structures at the skull base, including the orbital optic nerves, optic canal, cribriform plate, superior orbital fissure, foramen rotundum, foramen oval, internal auditory meatus, jugular foramen, and hypoglossal canal (Figure 1a,b).^12^ The spinal CTV (CTV_spinal_) included the entire subarachnoid space and extended laterally along the nerve roots, excluding the sacral nerve root canals. The inferior boundary was defined using MRI to identify the termination of the thecal sac (Figure 1c,d). The planning target volume (PTV) was generated by applying a 3 mm margin to the CTV_cranial_ and a 5 mm margin to the CTV_spinal_.

#### 2.2.2 Target in FP‐CSI

The delineation of the CTV in FP‐CSI was identical to that in S‐CSI. In addition, critical OARs were contoured. The hippocampi were delineated on T1‐weighted MRI according to the RTOG 0933 protocol, and a hippocampal avoidance region (HAR) was generated by expanding the hippocampal contour with a 5‐mm uniform margin.^13^ (Figure 1e,f). The hypothalamus and pituitary gland were delineated following established contouring guidelines, with a 5 mm expansion to define the HPA avoidance region (Figure 1g,h).^14^, ^15^ The vertebral contours included all the primary ossification centers and growth plates, adhering to the SIOPE Radiation Oncology Working Group (ROWG) recommendations (Figure 1i).^16^ The cochlea, a spiral‐shaped structure in the inner ear, was contoured based on the EPTN consensus atlas; the semicircular canals were excluded (Figure 1j).^15^ The scalp was defined as a 5‐mm thickness from the skin surface above the level of the foramen magnum, with optional exclusions of the area between the lateral canthus and below the orbital roof (Figure 1k,l).^17^ The PTV in FP‐CSI was generated by applying a 3 mm margin to the CTV_cranial_ and a 5 mm margin to the CTV_spinal_, excluding the HAR and HPA avoidance regions. Additional OARs delineated included the lenses, parotid and submandibular glands, thyroid, esophagus, heart, lungs, liver, kidneys, and intestines.

### 2.3 Dose prescription

The treatment prescription for the CSI PTV was set to deliver a total dose of 23.4 Gy in 13 fractions. All plans were normalized to ensure that 95% of the PTV received the prescribed dose. The acceptable compliance criteria for the PTV and constraints for OARs in S‐CSI and FP‐CSI are summarized in Table 1.

**TABLE 1:** Dose criteria for the PTV and constraints for OARs in S‐CSI and FP‐CSI. Dose prescription of 23.4 Gy in 13 fractions.

Structures	Dosimetric Parameters	S‐CSI	FP‐CSI
PTV	D_95%_ (Gy)	≥23.4	≥23.4
	D_2%_ (Gy)	≤25.7	≤25.7
	D_98%_ (Gy)	≥22	≥22
Hippocampus	D_0.03cc_ (Gy)	‐	≤16
	D_min_ (Gy)	‐	≤9
HPA	D_mean_ (Gy)	‐	≤16
Vertebrae	D_2%_ ‐ D_98%_(Gy)	‐	≤5
Cochlea	D_mean_ (Gy)	‐	≤21
Scalp	D_50%_	‐	≤19
Lenses	D_0.03cc_ (Gy)	≤7	≤7
Parotid	D_mean_ (Gy)	≤10	≤10
Submandibular gland	D_mean_ (Gy)	≤10	≤10
Thyroid	D_mean_ (Gy)	≤20	≤20
Esophagus	D_mean_ (Gy)	≤20	≤20
Heart	D_mean_ (Gy)	≤8	≤8
	V_15Gy_	≤5%	≤5%
	V_5Gy_	≤70%	≤70%
Lungs	D_mean_ (Gy)	≤8	≤8
	V_10Gy_	≤10%	≤10%
	V_5Gy_	≤60%	≤60%
Liver	D_mean_ (Gy)	≤8	≤8
	V_15Gy_	≤15%	≤15%
	V_5Gy_	≤75%	≤75%
Kidneys	D_mean_ (Gy)	≤7	≤7
	V_15Gy_	≤5%	≤5%
Intestines	D_mean_ (Gy)	≤8	≤8
	V_10Gy_	≤30%	≤30%

Abbreviations: S‐CSI, standard craniospinal irradiation; FP‐CSI, functional preservation craniospinal irradiation; HPA, hypothalamic‐pituitary axis.

The dose criteria for the hippocampus, HPA, vertebrae, cochlea, and scalp in FP‐CSI (Table 1) were based on established dose–toxicity evidence. Hippocampal constraints followed RTOG 0933.^13^ For the HPA, the threshold of approximately 16 Gy was adopted from Merchant et al., who reported a 50% risk of growth hormone deficiency at this dose level.^18^ The vertebral homogeneity requirement was derived from the SIOPE‐ROWG consensus, which emphasize minimizing anterior–posterior dose gradients to reduce long‐term deformity.^16^ The scalp limits were informed by reported dose–response associations with severe permanent alopecia.^6^ The cochlear constraint of 21 Gy was selected to avoid focal hotspots while preserving coverage of the internal auditory canal, which lies adjacent to the cochlea and is included in the CTV. These evidence‐based considerations motivated the incorporation of functional‐sparing objectives into FP‐CSI.

### 2.4 Treatment planning

The treatment plans were optimized using the Monaco treatment planning system (version 6.00.11, Elekta AB, Stockholm, Sweden) and delivered with 6 MV photon beams from a linear accelerator (Infinity linac, Elekta AB, Stockholm, Sweden). The linac was equipped with 80 pairs of multi‐leaf collimators (5 mm leaf width at the isocenter) and the maximum dose rate was set to 600 MU/min. Dose calculations were performed using the Monte Carlo algorithm with a 0.2 cm calculation grid and 1% uncertainty.

**FIGURE 2:**
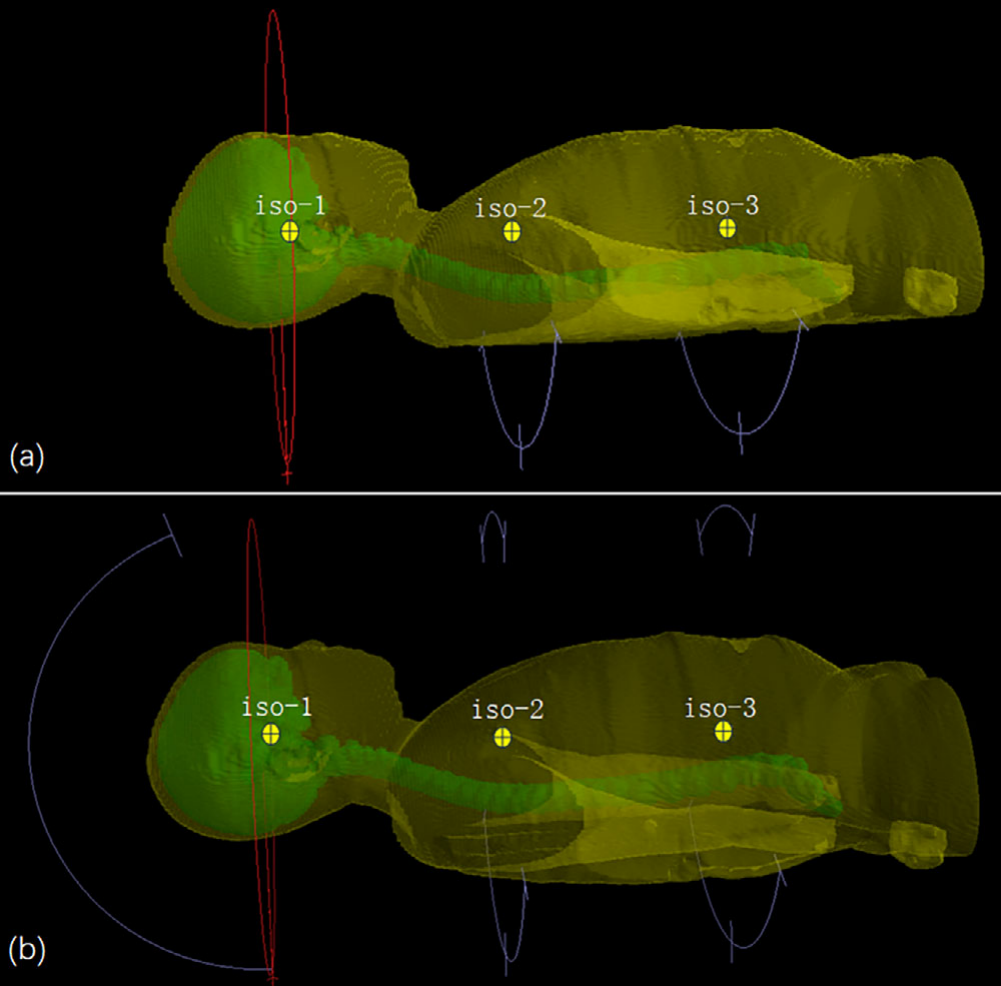
Schematic diagrams illustrating the isocenter and beam arrangements for S‐CSI and FP‐CSI. (a) In S‐CSI, two full arcs with collimator angles of 30° and 330° were employed at iso‐1 and partial arcs with gantry angles of 180°–120° and 240°–181° were used at iso‐2 and iso‐3. (b) In FP‐CSI, an additional non‐coplanar VMAT arc of 160° with a couch angle of 90° was introduced at iso‐1. At iso‐2 and iso‐3, partial arcs spanning gantry angles from 30° to 330°, with collimator angle of 0°, were additionally employed.

Beam arrangements were determined based on the length of the CSI PTV and consisted of two or three beam sets to cover the entire target volume. The isocenter (iso‐1) of the first set was positioned at the level of the cranial PTV, while the second and third sets were placed at the upper spinal PTV (iso‐2) and lower spinal PTV (iso‐3), respectively. These three isocenters were aligned collinearly, requiring only a longitudinal couch shift for transitions (Figure 2).

For the cranial PTV in the S‐CSI plan, two full arcs with collimator angles of 30° and 330° were employed to ensure target coverage while sparing the lenses. For the spinal PTV, partial arcs with gantry angles of 180°–120° and 240°–181° were used, with collimator angles of 10° and 350° (Figure 2a). These beam arrangements effectively reduced OAR doses and avoided entry through the shoulders and arms. To improve robustness at field junctions, a staggered overlap technique was applied, with overlap regions of 6–9 cm.^19^ In this approach, the field edges of adjacent arc sets were intentionally shifted in an appropriate step size to distribute the longitudinal dose gradient and prevent sharp hot or cold spots. When the spinal PTV length was <40 cm, the third beam set was omitted.

In the FP‐CSI plan, an additional non‐coplanar VMAT arc (gantry 160°, couch 90°, collimator 0°) was introduced at iso‐1. This arc was chosen to enhance sparing of key functional structures (particularly the hippocampus and HPA) by providing oblique beam paths that cannot be achieved with coplanar arcs alone. The non‐coplanar geometry increased the angular diversity available to the optimizer, enabling improved modulation around cranial OARs while maintaining adequate target coverage. At iso‐2 and iso‐3, partial arcs spanning gantry angles from 30° to 330°, with a collimator angle of 0°, were additionally employed (Figure 2b). VMAT inverse planning objectives were prioritized as follows: the primary objective was to reduce vertebral dose gradients and spare the hippocampus, HPA, scalp, and cochlea; the secondary objective was to achieve adequate CSI PTV coverage with acceptable homogeneity; and the tertiary objective was to further minimize doses to other OARs. The dose objectives listed in Table 1 correspond to the use of Target Penalty, Underdose DVH, Maximum Dose, and Parallel cost functions within the optimization process. Automatic weight adjustment in Monaco was employed throughout planning to dynamically balance these objectives, ensuring that PTV dose requirements were achieved while maintaining optimal OAR sparing. By contrast, the S‐CSI plan included no explicit constraints for the hippocampus, HPA, scalp, cochlea, or vertebrae, with priority placed on ensuring CSI PTV coverage while reducing OAR doses only where feasible.

### 2.5 Treatment planning evaluation

The evaluated dosimetric parameters for the PTV included D_2%_, D_98%_, homogeneity index (HI), and Paddick conformity index (CI):

(1) $$HI=\frac{D_{2\%}-D_{98\%}}{D_{50\%}}\;$$

where D_50%_ represents the median dose to the PTV, while D_2%_ and D_98%_ denote the near‐maximum and near‐minimum doses that cover 2% and 98% of the PTV volume, respectively, as indicated by the dose‐volume histogram (DVH). HI values approaching 0 indicate superior dose homogeneity.

(2) $$CI=\frac{V_{t,ref}^{2}}{V_{t}\times V_{ref}}\;$$

where $V_{t}$ represents the total volume of the PTV, $V_{t,ref}$ is the volume of the target covered by the prescription dose, and $V_{ref}$ is the total volume of the prescription dose. The CI value ranges from 0 to 1, with values closer to 1 indicating better conformity.

The dosimetric parameters for OARs were also extracted for comparison. These included the minimum, near‐maximum dose (D_0.03cc_), and mean doses to the hippocampus; the D_0.03cc_ to the lenses; the median dose to the scalp; the dose gradient to the vertebrae; and the mean doses to the hypothalamus, pituitary gland, cochlea, and other OARs. The dose gradient to the vertebrae was defined as D_2%_‐D_98%_.

### 2.6 Normal tissue complication probability (NTCP) modeling

Biological models were applied to estimate the probability of normal tissue complications and to compare radiobiological effects between FP‐CSI and S‐CSI plans. The normal tissue complication probability (NTCP) was calculated using the Lyman–Kutcher–Burman (LKB) model or multivariate parametric models, depending on the organ of interest.

In the LKB model, NTCP is defined as:

(3) $$NTCP=\frac{1}{\sqrt{2\pi }}\;\int_{-\infty }^{t}\exp\left(-\frac{x^{2}}{2}\right)dx$$

(4) $$t=\frac{gEUD-TD_{50}}{m\cdot TD_{50}}\;$$

where gEUD is the generalized equivalent uniform dose, calculated as:

(5) $$gEUD={\left(\sum_{i}v_{i}D_{i}^{\frac{1}{n}}\right)}^{n}\;$$

here, $v_{i}$ is the relative subvolume receiving a dose $D_{i}$. $TD_{50}$ represents the tolerance dose associated with a 50% risk of complication for the whole organ. n describes the volume effect and m defines the slope of the dose–response curve at $TD_{50}$.

#### 2.6.1 Neurocognitive impairment

Neurocognitive function impairment related to hippocampal irradiation was estimated using the LKB model, as proposed by Gondi et al.^20^ The equivalent dose in 2‐Gy fractions (EQD_2_) to 40% of the hippocampal volume was used in place of gEUD:

(6) $$t=\frac{EQD_{2}\left(D_{40\%}\right)-TD_{50}}{m\cdot TD_{50}}\;$$

the model parameters were $TD_{50}$= 14.88Gy and m=0.54, consistent with the values reported by Gondi et al.^20^

#### 2.6.2 Alopecia

Acute radiation induced grade 2 alopecia was evaluated based on the scalp dose using the LKB model, with organ‐specific parameters derived from Palma et al.^21^ The corresponding $TD_{50}$, m, and n values are summarized in Table 2.

**TABLE 2:** NTCP model parameters for functional organs‐at‐risk.

Organs at risk	Complication	Model type	Model parameters	Reference
Hippocampus	Neurocognitive impairment	LKB	TD_50_ = 14.88Gy m = 0.54 α/β = 2	Gondi et al., 2012^20^
Scalp	Acute grade 2 radiation‐induced alopecia	LKB	TD_50_ = 22Gy m = 0.54 n = 0.14	Palma et al, 2020^21^
Hypothalamus/Pituitary	Endocrine dysfunction	Multivariate parametric model	𝛾 = 0.56 const = 3.13 β_age_ = −0.106 β_age_^2^ = 0.007 β_dose_ = −0.049 t_i_ = 5	Vatner et al, 2018^22^
Cochlea	Hearing loss	Multivariate logistic regression model	b_0_ = −5.3 b_1_ = 0.085	Murphy et al, 2024^23^

Abbreviations: LKB, Lyman–Kutcher–Burman model; TD_50_, tolerance dose associated with a 50% risk of complication for the whole organ.

#### 2.6.3 Endocrine dysfunction

Endocrine dysfunction was modeled using a multivariate parametric model, incorporating hypothalamic–pituitary median dose, patient age, and follow‐up time, as described by Vatner et al.^22^ The NTCP was calculated as:

(7) $$NTCP=1-\frac{1}{1+{\left(\varphi_{i}\cdot t_{i}\right)}^{\frac{1}{\gamma }}}$$

(8) $$\begin{array}{cc} \varphi_{i} & =\exp\left(-\left(const+\beta_{age}\cdot age_{i}+\beta_{age^{2}}\cdot ag{e_{i}}^{2}\right.\right.\\ & \;\left.\left.+\;\beta_{dose}\cdot dose\right)\right)\; \end{array}$$

where $t_{i}$ represents the post‐treatment follow‐up time and 5 is used in this study. β coefficients describe the respective effects of age and radiation dose. The dose is the mean of the D_50%_ of the hypothalamus and pituitary. The corresponding parameters are summarized in Table 2.

#### 2.6.4 Hearing loss

Radiation induced hearing loss was estimated using the multivariate logistic regression model recommended by Murphy et al.^23^ The risk was calculated as:

(9) $$NTCP=\frac{\exp\left(b_{0}+b_{1}\cdot dose\right)}{1+\exp\left(b_{0}+b_{1}\cdot dose\right)}\;$$

where dose represents the median cochlear dose, b_0_ and b_1_ are the fitted logistic coefficients, provided in Table 2.

### 2.7 Plan robustness

To assess the robustness of the treatment plans against patient setup uncertainties, thirty perturbed dose distributions were recalculated for both FP‐CSI and S‐CSI plans. The perturbations simulated setup errors, including 18 translational shifts of ±2, ±3, and ±5 mm along the superior–inferior (SI), left–right (LR), and anterior–posterior (AP) axes, and twelve rotational errors in pitch, yaw, and roll of ±1.0° and ±2.0°.

For each uncertainty scenario, a DVH was generated, and the ensemble of these DVHs formed a DVH band representing the range of dose variations under all perturbations. The band width (defined as the difference between the maximum and minimum values of a given DVH metric across scenarios) was used as a quantitative indicator of plan robustness.

To facilitate comparison between FP‐CSI and S‐CSI, the robustness index (λ) was normalized to the nominal dose value and calculated as:

(10) $$\lambda_{x}=\left|\frac{D_{x}^{best}-D_{x}^{worst}}{D_{x}^{nominal}}\right|\;\cdot 100\%$$

where $\lambda_{x}$ represents the robustness index, $D_{x}^{best}$ and $D_{x}^{worst}$ denote the highest and lowest dose values observed among the uncertainty scenarios, and $D_{x}^{nominal}$ is the corresponding dose in the nominal (unperturbed) plan.

The DVH indices used for evaluation were D_95%_ for the PTV, D_40%_ for the hippocampus, and D_50%_ for the HPA, scalp, and cochlea. A smaller λ value indicates higher robustness against setup uncertainties.

### 2.8 Plan complexity and plan QA

Plan complexity and plan QA were evaluated to comprehensively assess the deliverability and accuracy of the FP‐CSI and S‐CSI plans. The complexity of each treatment plan was quantified using multiple metrics, including monitor units (MU), the number of control points (CPs), plan‐averaged beam irregularity (PI),^24^ modulation complexity score (MCS),^25^ small aperture score (10 mm) (SAS10),^26^ mean field area (MFA),^26^ mean leaf gap (MLG),^27^ and mean leaf travel (MLT)^25^ (definitions and equations are provided in the supplementary material Equations S1–S15). Generally, higher MU, larger number of CPs, higher PI, higher SAS10, and greater MLT indicate increased modulation and plan complexity. Larger MFA and MLG represent simpler field shapes and lower complexity. The MCS is inversely correlated with complexity, with lower values indicating higher modulation. To provide a detailed evaluation, the complexity analysis was performed for both the overall plan and separately for the cranial (iso‐1) and spinal (iso‐2/iso‐3) beams.

Measurement‐based pretreatment QA was performed using a two‐dimensional ion chamber array detector, MatriXX (IBA, Schwarzenbruck, Germany). Verification plans for each isocenter were calculated on the MatriXX phantom. For beam lengths exceeding the 24 cm measurement region, the inferior section was measured in the standard orientation, while the superior section was measured with the MatriXX rotated 180° to avoid exposing its electronics. The measured and planned dose distributions were compared using 2D global gamma analysis for each isocenter, evaluated with the OmniPro‐I'mRT software (IBA, Schwarzenbruck, Germany) at criteria of 3%/3 mm and 2%/2 mm.

Log file–based three‐dimensional dose reconstruction was additionally performed to evaluate volumetric dose deviations between the delivered and planned doses. The commercial GPU‐accelerated Monte Carlo dose engine ArcherQA (Wisdom Technology Co., Ltd., Hefei, China) was used for this purpose.^28^ This system utilizes the linear accelerator delivery log files to reconstruct the actual beam parameters and regenerate a modified RT Plan, from which the delivered 3D dose is recalculated on the patient's CT dataset. The reconstructed dose distributions were compared with the planned doses by analyzing DVH‐based dose deviations (ΔD_x_), defined as the difference between delivered and planned values of specific DVH metrics. The evaluated parameters included ΔD_95%_, ΔD_98%_, ΔD_2%_, and ΔD_mean_ for the PTV, ΔD_40%_ for the hippocampus, and ΔD_50%_ for the HPA, scalp, and cochlea.

### 2.9 Setup and image‐guided protocol

For CSI delivery, patient immobilization during treatment was identical to that used during CT simulation. Image guidance was performed prior to each fraction. For the first fraction, all isocenters underwent verification before irradiation. Cone beam computed tomography (CBCT) was first acquired at the cranial isocenter, and the corresponding couch correction was applied. The couch was then shifted to the lower spinal isocenter for a second CBCT acquisition. Setup was accepted if the residual deviation was less than 3 mm; otherwise, the patient was repositioned. A subsequent kV orthogonal image pair was obtained at the upper spinal isocenter, with an acceptance threshold of 3 mm.

For the second and third fractions, kV orthogonal imaging was first performed at the cranial isocenter. Deviations >2 mm required patient repositioning, otherwise, couch correction was applied. The couch was then moved to the lower and upper spinal isocenters sequentially, each verified with orthogonal imaging and a 3 mm tolerance. Repositioning was performed if deviations exceeded tolerance at any isocenter.

For the remaining fractions, full verification of all isocenters was performed once per week using the same workflow as above. On the other treatment days, patients were positioned to cranial setup marks, and daily verification was performed at the lower spinal isocenter using kV orthogonal imaging. Repositioning was required for deviations >3 mm.

### 2.10 Statistical analyses

Statistical comparisons between FP‐CSI and S‐CSI plans were performed using paired *t*‐tests. Normality was assessed using the Shapiro–Wilk test. Given the limited sample size (*n* = 8), non‐parametric Wilcoxon signed‐rank tests were additionally performed to confirm the robustness of the findings. Results are reported as mean ± standard deviation, with *p*‐values < 0.05 considered statistically significant. All analyses were conducted using SPSS software (version 26.0, IBM, USA).

## 3 RESULTS

### 3.1 Dosimetry comparisons

**FIGURE 3:**
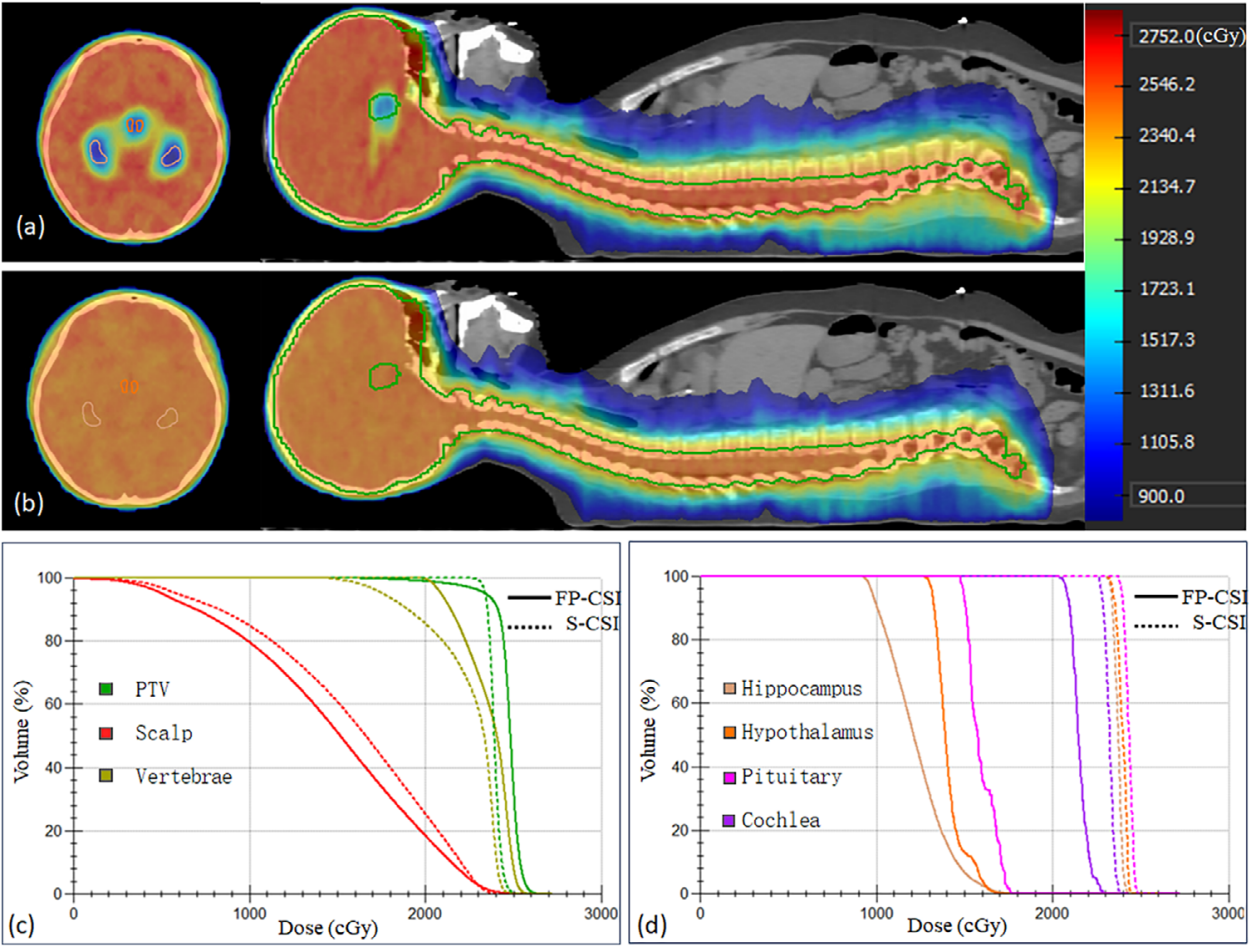
Representative dose distribution and DVH comparisons for FP‐CSI and S‐CSI. (a) Dose distribution of the FP‐CSI plan. (b) Dose distribution of the S‐CSI plan. (c) DVH comparison for the PTV, scalp, and vertebrae. (d) DVH comparison for critical structures, including the hippocampus, hypothalamus, pituitary, and cochlea. DVH, dose‐volume histogram; FP‐CSI, functional preservation craniospinal irradiation; S‐CSI, standard craniospinal irradiation. Dose values in the DVH are displayed in cGy due to system‐setting; all reported dose metrics in the manuscript are expressed in Gy for consistency.

The dose distribution and DVH for FP‐CSI and S‐CSI are illustrated in Figure 3. Quantitative comparisons of the two techniques are summarized in Table 3. For the PTV, FP‐CSI exhibited a lower D_98%_ (21.7 ± 0.4 Gy) compared to S‐CSI (23.2 ± 0.1 Gy, *p* < 0.01), and the D_2%_ was higher in FP‐CSI (25.5 ± 0.2 Gy vs. 24.8 ± 0.1 Gy, *p* < 0.01). The HI and CI of the PTV in FP‐CSI demonstrated slight inferiority compared to S‐CSI (HI: 0.16 vs. 0.07, *p *< 0.01; CI: 0.88 vs. 0.93, *p *< 0.01).

**TABLE 3:** Dose–volume parameters for the PTV and OARs in the FP‐CSI and S‐CSI plans.

Structures	Dosimetric Parameters	FP‐CSI	S‐CSI	*p*‐value
PTV	D_2%_ (Gy)	25.5 ± 0.2	24.8 ± 0.1	<0.01
	D_98%_ (Gy)	21.7 ± 0.4	23.2 ± 0.1	<0.01
	HI	0.16 ± 0.02	0.07 ± 0.003	<0.01
	CI	0.88 ± 0.02	0.93 ± 0.02	<0.01
PTV_cranial_	D_2%_ (Gy)	25.6 ± 0.2	24.8 ± 0.1	<0.01
	D_95%_ (Gy)	23.1 ± 0.1	23.4 ± 0.03	<0.01
	D_98%_ (Gy)	21.2 ± 0.5	23.2 ± 0.1	<0.01
Hippocampus	D_0.03cc_(Gy)	16.5 ± 0.5	24.4 ± 0.1	<0.01
	D_min_(Gy)	9.3 ± 0.4	23.1 ± 0.1	<0.01
	D_mean_(Gy)	12.4 ± 0.2	23.9 ± 0.1	<0.01
Hypothalamus	D_mean_(Gy)	14.7 ± 0.5	23.9 ± 0.2	<0.01
Pituitary	D_mean_(Gy)	15.4 ± 0.6	24.1 ± 0.2	<0.01
Vertebrae	D_2%_‐D_98%_(Gy)	4.7 ± 0.2	8.7 ± 1.9	<0.01
Cochlea	D_mean_(Gy)	21.4 ± 0.2	23.1 ± 0.3	<0.01
Scalp	D_50%_ (Gy)	15.3 ± 2.0	16.4 ± 1.9	<0.01
Lenses	D_0.03cc_(Gy)	6.1 ± 0.3	5.4 ± 0.1	<0.01
Parotid	D_mean_(Gy)	10.0 ± 0.7	11.1 ± 1.1	<0.01
Submandibular gland	D_mean_(Gy)	9.7 ± 1.1	9.1 ± 1.3	<0.01
Thyroid	D_mean_(Gy)	15.3 ± 1.6	14.3 ± 1.7	<0.01
Esophagus	D_mean_(Gy)	16.8 ± 0.6	14.4 ± 0.9	<0.01
Heart	D_mean_(Gy)	6.3 ± 0.4	5.7 ± 0.5	<0.01
Lungs	D_mean_(Gy)	5.3 ± 0.8	4.7 ± 0.9	<0.01
Liver	D_mean_(Gy)	5.2 ± 0.5	4.6 ± 0.6	<0.01
Kidneys	D_mean_(Gy)	6.1 ± 1.2	4.4 ± 1.3	<0.01
Intestines	D_mean_(Gy)	6.5 ± 1.2	5.7 ± 1.1	<0.01

Abbreviations: FP‐CSI, functional preservation craniospinal irradiation; S‐CSI, standard craniospinal irradiation.

To further examine the regional coverage performance, the cranial portion of the target (PTV_cranial_) was analyzed separately. The D_95%_ of PTV_cranial_ for FP‐CSI was 23.1 Gy, corresponding to 98.7% of the prescribed dose, while the D_98%_ exceeded 90% of the prescribed dose, both within clinically acceptable limits.

FP‐CSI achieved significant dose reductions to critical structures, particularly the hippocampus and HPA. The mean dose (D_mean_) to the hippocampus decreased from 23.9 Gy in S‐CSI to 12.4 Gy in FP‐CSI, while the hypothalamus and pituitary gland experienced reductions from 23.9 Gy and 24.1 Gy in S‐CSI to 14.7 Gy and 15.4 Gy, respectively (*p* < 0.01 for all comparisons). FP‐CSI also improved vertebral dose homogeneity, lowering the dose gradient (D_2%_–D_98%_) from 8.7 Gy in S‐CSI to 4.7 Gy. Additionally, FP‐CSI moderately reduced the doses to the cochlea and scalp. Detailed results are provided in Table 3.

For other OARs, FP‐CSI generally maintained comparable or slightly elevated mean doses relative to those of the S‐CSI. Notably, FP‐CSI resulted in higher maximum doses to the lenses (6.1 Gy vs. 5.4 Gy) but reduced the mean doses to the parotid glands (10.0 Gy vs. 11.1 Gy). However, mean dose differences for other OARs, including the submandibular glands, thyroid, esophagus, heart, lungs, liver, kidneys, and intestines, were generally minor, with increases of less than 1 Gy in FP‐CSI compared to S‐CSI. Detailed dosimetric data are presented in Table 3.

### 3.2 NTCP evaluation

The NTCP results derived from the biological models and the corresponding dosimetric parameters are summarized in Table 4. Overall, FP‐CSI demonstrated lower NTCP values than S‐CSI across all evaluated OARs.

**TABLE 4:** Comparison of predicted NTCP values and corresponding dosimetric parameters for FP‐CSI and S‐CSI.

Structures	Dosimetric/biological Parameters	FP‐CSI	S‐CSI	*p*‐value
Hippocampus	D_40%_(Gy)	12.7 ± 0.2	24.0 ± 0.1	<0.01
	EQD_2_(D_40_) (Gy)	9.4 ± 0.2	23.0 ± 0.1	<0.01
	NTCP (%)	24.9 ± 0.6	84.5 ± 0.4	<0.01
Hypothalamus Pituitary	D_50%_(Gy)	14.6 ± 0.5	23.9 ± 0.2	<0.01
	D_50%_(Gy)	15.2 ± 0.6	24.1 ± 0.2	<0.01
	NTCP (%)	27.3 ± 8.4	44.7 ± 11.3	<0.01
Cochlea	D_50%_(Gy)	21.4 ± 0.2	23.1 ± 0.3	<0.01
	NTCP (%)	3.0 ± 0.04	3.4 ± 0.1	<0.01
Scalp	gEUD(Gy)	18.0 ± 1.4	18.5 ± 1.3	<0.01
	NTCP (%)	36.8 ± 4.4	38.3 ± 4.1	<0.01

Abbreviations: EQD_2_: equivalent dose in 2‐Gy fractions; FP‐CSI, functional preservation craniospinal irradiation; gEUD: generalized equivalent uniform dose; NTCP, normal tissue complication probability; S‐CSI, standard craniospinal irradiation.

The predicted risk of neurocognitive impairment associated with hippocampal irradiation decreased markedly from 84.5 ± 0.4% in S‐CSI to 24.9 ± 0.6% in FP‐CSI. For the HPA, the NTCP for endocrine dysfunction was reduced from 44.7 ± 11.3% to 27.3 ± 8.4%. The cochlear NTCP for hearing loss slightly declined from 3.4 ± 0.1% to 3.0 ± 0.04%, and the scalp NTCP for acute grade 2 alopecia decreased from 38.3 ± 4.1% to 36.8 ± 4.4%.

### 3.3 Plan robustness

**FIGURE 4:**
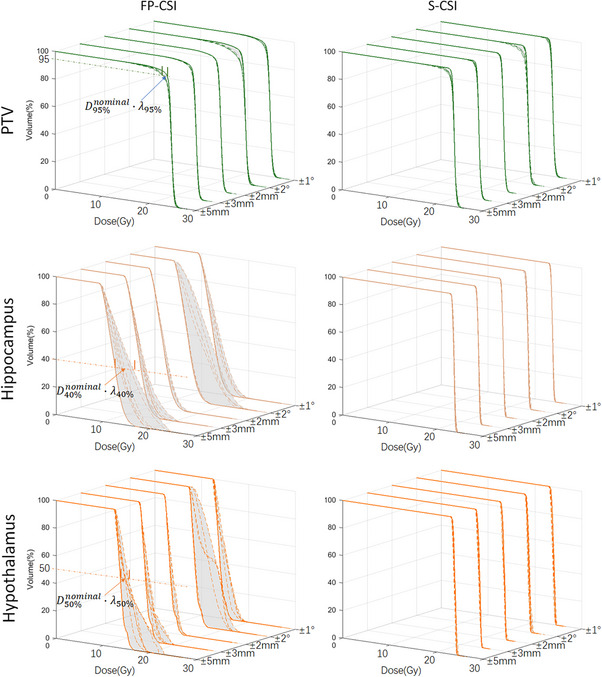
The DVH bands and corresponding robustness index (λ) of the PTV, hippocampus and hypothalamus under various setup and rotational perturbations for FP‐CSI and S‐CSI plans.

**FIGURE 5:**
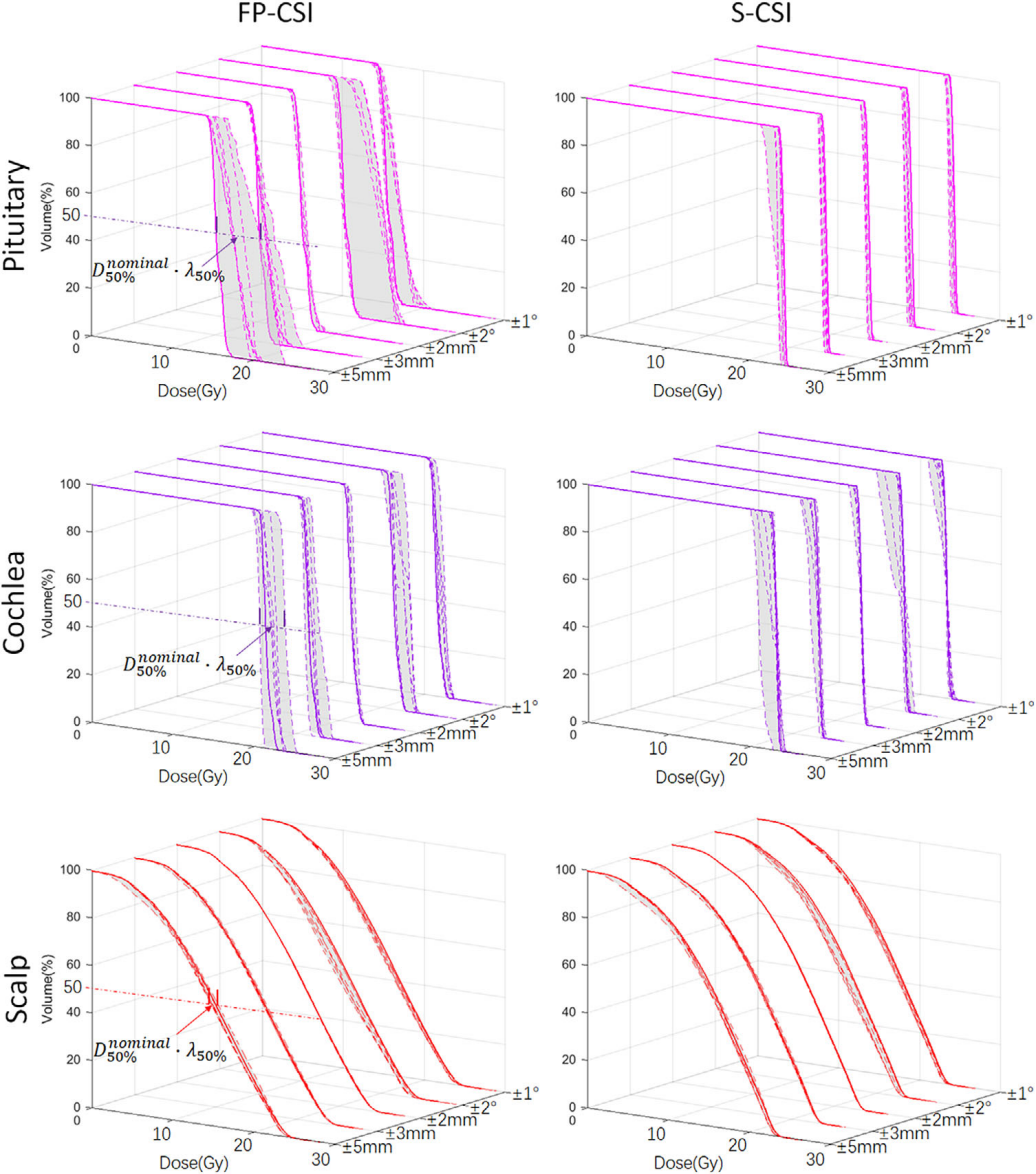
The DVH bands and corresponding robustness index (λ) of the pituitary, cochlea and scalp under various setup and rotational perturbations for FP‐CSI and S‐CSI plans.

Figures 4 and 5 illustrates the DVH bands of the PTV and functional OARs under various setup and rotational perturbations for FP‐CSI and S‐CSI plans, while the corresponding quantitative robustness indices (λ) are summarized in Table 5. Across all evaluated uncertainties, FP‐CSI demonstrated larger λ_x_ compared with S‐CSI, indicating lower robustness to both translational and rotational setup errors.

**TABLE 5:** Robustness index (λ) for FP‐CSI and S‐CSI plans under setup and rotational uncertainties.

Structures	Uncertainty	Robustness index	FP‐CSI	S‐CSI	*p*‐value
PTV	±2mm	λ_95%_ (%)	0.8 ± 0.04	0.7 ± 0.04	<0.01
	±3mm	λ_95%_ (%)	1.7 ± 0.1	1.3 ± 0.2	<0.01
	±5mm	λ_95%_ (%)	5.4 ± 0.2	3.4 ± 0.7	<0.01
	±1.0°	λ_95%_ (%)	3.1 ± 0.9	1.4 ± 0.1	<0.01
	±2.0°	λ_95%_ (%)	10.8 ± 2.3	6.4 ± 1.7	<0.01
Hippocampus	±2mm	λ_40%_ (%)	3.0 ± 0.1	0.7 ± 0.04	<0.01
	±3mm	λ_40%_ (%)	8.1 ± 0.8	1.3 ± 0.2	<0.01
	±5mm	λ_40%_ (%)	34.6 ± 3.2	3.4 ± 0.7	<0.01
	±1.0°	λ_40%_ (%)	13.9 ± 2.4	1.4 ± 0.1	<0.01
	±2.0°	λ_40%_ (%)	45.1 ± 3.9	6.4 ± 1.7	<0.01
Hypothalamus	±2mm	λ_50%_ (%)	3.2 ± 0.1	0.7 ± 0.04	<0.01
	±3mm	λ_50%_ (%)	5.6 ± 0.4	1.3 ± 0.2	<0.01
	±5mm	λ_50%_ (%)	20.8 ± 8.4	3.4 ± 0.7	<0.01
	±1.0°	λ_50%_ (%)	13.5 ± 3.9	1.4 ± 0.1	<0.01
	±2.0°	λ_50%_ (%)	42.2 ± 9.8	6.4 ± 1.7	<0.01
Pituitary	±2mm	λ_50%_ (%)	4.5 ± 1.1	0.7 ± 0.04	<0.01
	±3mm	λ_50%_ (%)	16.6 ± 5.2	1.3 ± 0.2	<0.01
	±5mm	λ_50%_ (%)	47.1 ± 12.2	3.4 ± 0.7	<0.01
	±1.0°	λ_50%_ (%)	13.5 ± 2.9	1.4 ± 0.1	<0.01
	±2.0°	λ_50%_ (%)	40.4 ± 7.6	6.4 ± 1.7	<0.01
Cochlea	±2mm	λ_50%_ (%)	3.1 ± 0.6	0.7 ± 0.04	<0.01
	±3mm	λ_50%_ (%)	8.4 ± 0.4	1.3 ± 0.2	<0.01
	±5mm	λ_50%_ (%)	15.1 ± 0.4	3.4 ± 0.7	<0.01
	±1.0°	λ_50%_ (%)	5.5 ± 0.7	1.4 ± 0.1	<0.01
	±2.0°	λ_50%_ (%)	10.6 ± 1.1	6.4 ± 1.7	<0.01
Scalp	±2mm	λ_50%_ (%)	1.0 ± 0.1	0.7 ± 0.04	<0.01
	±3mm	λ_50%_ (%)	5.6 ± 2.6	1.3 ± 0.2	<0.01
	±5mm	λ_50%_ (%)	11.1 ± 5.5	3.4 ± 0.7	<0.01
	±1.0°	λ_50%_ (%)	4.7 ± 0.1	1.4 ± 0.1	<0.01
	±2.0°	λ_50%_ (%)	9.1 ± 1.5	6.4 ± 1.7	<0.05

Abbreviations: FP‐CSI, functional preservation craniospinal irradiation; S‐CSI, standard craniospinal irradiation.

For the PTV, FP‐CSI consistently showed larger λ_95%_ values than S‐CSI under both translational and rotational uncertainties. At ±2 mm, λ_95%_ increased from 0.7% for S‐CSI to 0.8% for FP‐CSI, and at ±5 mm from 3.4% to 5.4%. Similarly, rotational errors led to greater deviations in FP‐CSI, with λ_95%_ increasing from 1.4% (S‐CSI) to 3.1% (FP‐CSI) at ±1.0° and from 6.4% to 10.8% at ±2.0° (all *p* < 0.01). These results indicate that S‐CSI provides superior dose stability compared with FP‐CSI under positional perturbations.

Similar trends were observed for the hippocampus, hypothalamus, pituitary, cochlea, and scalp, where FP‐CSI showed significantly higher λ values than S‐CSI (*p* < 0.01). The largest differences were seen in the hippocampus and hypothalamic–pituitary regions, with FP‐CSI λ exceeding 40% under ±2.0° rotations, compared to below 7% for S‐CSI.

### 3.4 Plan complexity and plan QA

**FIGURE 6:**
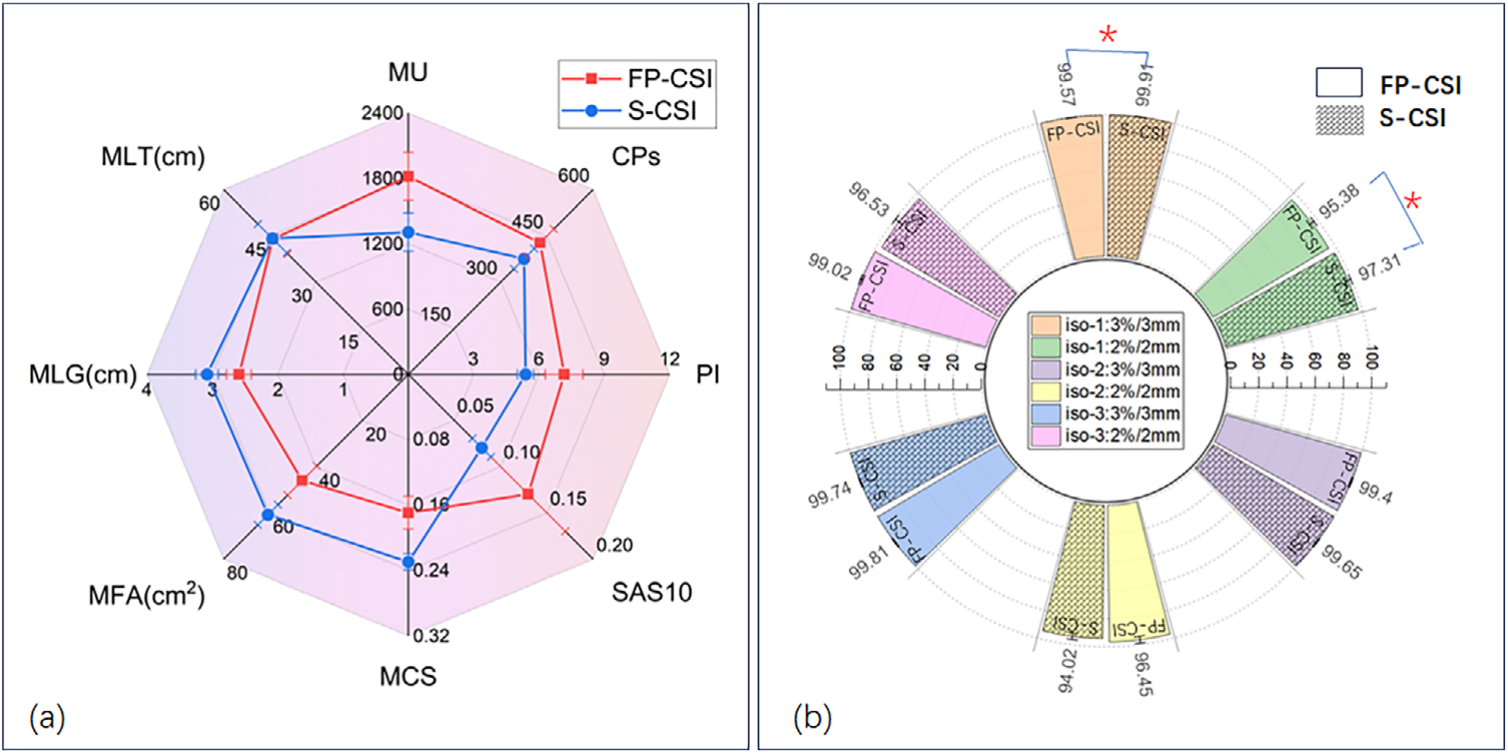
Comparison of plan complexity and quality assurance results between FP‐CSI and S‐CSI. (a) Complexity metrics: FP‐CSI plans are represented by the red line, and S‐CSI plans by the blue line. (b) Gamma passing rates for each isocenter across the two techniques. * represents a statistically significant difference. FP‐CSI, functional preservation craniospinal irradiation; S‐CSI, standard craniospinal irradiation.

The complexity metrics for the FP‐CSI and S‐CSI plans are compared in Figure 6a. Overall, FP‐CSI plans demonstrated higher modulation complexity, as reflected by increased MU, CPs, PI, and SAS10 values, and by decreased MCS, MFA, and MLG values compared to S‐CSI plans.

When complexity was analyzed separately for the cranial (iso‐1) and spinal (iso‐2/iso‐3) beams (Table 6), similar trends were observed in the cranial region, where FP‐CSI showed significantly higher MU, CPs, PI, and SAS10, together with lower MCS, MFA, and MLG values compared with S‐CSI (all *p* < 0.01). These findings confirm that FP‐CSI plans for cranial targets were substantially more modulated and complex than S‐CSI plans. In contrast, for the spinal beams, most complexity metrics showed no statistically significant differences between the two techniques (*p* > 0.05), suggesting comparable delivery efficiency in the spinal region. The increased overall complexity of FP‐CSI therefore mainly originated from the cranial field design rather than the spinal components.

**TABLE 6:** Comparison of beam complexity metrics between FP‐CSI and S‐CSI for cranial and spinal PTVs.

Complexity metric	Beams for PTV_cranial_			Beams for PTV_spinal_		
	FP‐CSI	S‐CSI	*p*‐value	FP‐CSI	S‐CSI	*p*‐value
CPs	319.3 ± 19.2	267.3 ± 13.1	<0.01	108.4 ± 28.6	108.5 ± 28.7	0.35
MU	1291.9 ± 129.8	799.8 ± 40.7	<0.01	524.2 ± 160.4	506.7 ± 165.1	0.05
PI	8.3 ± 1.0	5.8 ± 0.5	<0.01	4.9 ± 1.1	4.8 ± 0.7	0.94
MCS	0.1 ± 0.02	0.2 ± 0.01	<0.01	0.3 ± 0.02	0.3 ± 0.03	0.06
SAS10	0.2 ± 0.05	0.1 ± 0.02	<0.01	0.04 ± 0.01	0.06 ± 0.03	0.20
MFA (cm^2^)	33.3 ± 4.7	54.6 ± 3.1	<0.01	78.4 ± 18.0	74.6 ± 19.3	0.12
MLG (cm)	2.3 ± 0.3	3.3 ± 0.2	<0.01	2.9 ± 0.2	2.8 ± 0.2	0.08
MLT (cm)	109.3 ± 8.9	111.9 ± 7.5	0.18	14.1 ± 0.6	13.2 ± 0.6	0.06

Abbreviations: FP‐CSI, functional preservation craniospinal irradiation; MCS, modulation complexity score; MFA, mean field area; MLG, mean leaf gap; MLT, mean leaf travel; PI, plan‐averaged beam irregularity; SAS10, small aperture score (10 mm); S‐CSI, standard craniospinal irradiation.

The gamma passing rates for the FP‐CSI and S‐CSI plans at each isocenter are shown in Figure 6b. For iso‐1 beams, FP‐CSI plans achieved average gamma passing rates of 99.6% ± 0.2% and 95.4% ± 1.9% at the 3%/3 mm and 2%/2 mm criteria, respectively. These were slightly lower than the corresponding rates for S‐CSI plans (99.9% ± 0.1% at 3%/3 mm and 97.3% ± 2.1% at 2%/2 mm), with statistically significant differences (*p* < 0.05). For iso‐2 and iso‐3 beams, gamma passing rates for both techniques exceeded 99% at the 3%/3 mm criteria and 94% at the 2%/2 mm criteria, With no statistically significant differences. Notably, the slightly reduced gamma passing rates of FP‐CSI for the cranial beams were consistent with its higher modulation complexity, indicating an expected correlation between plan complexity and deliverability.

**FIGURE 7:**
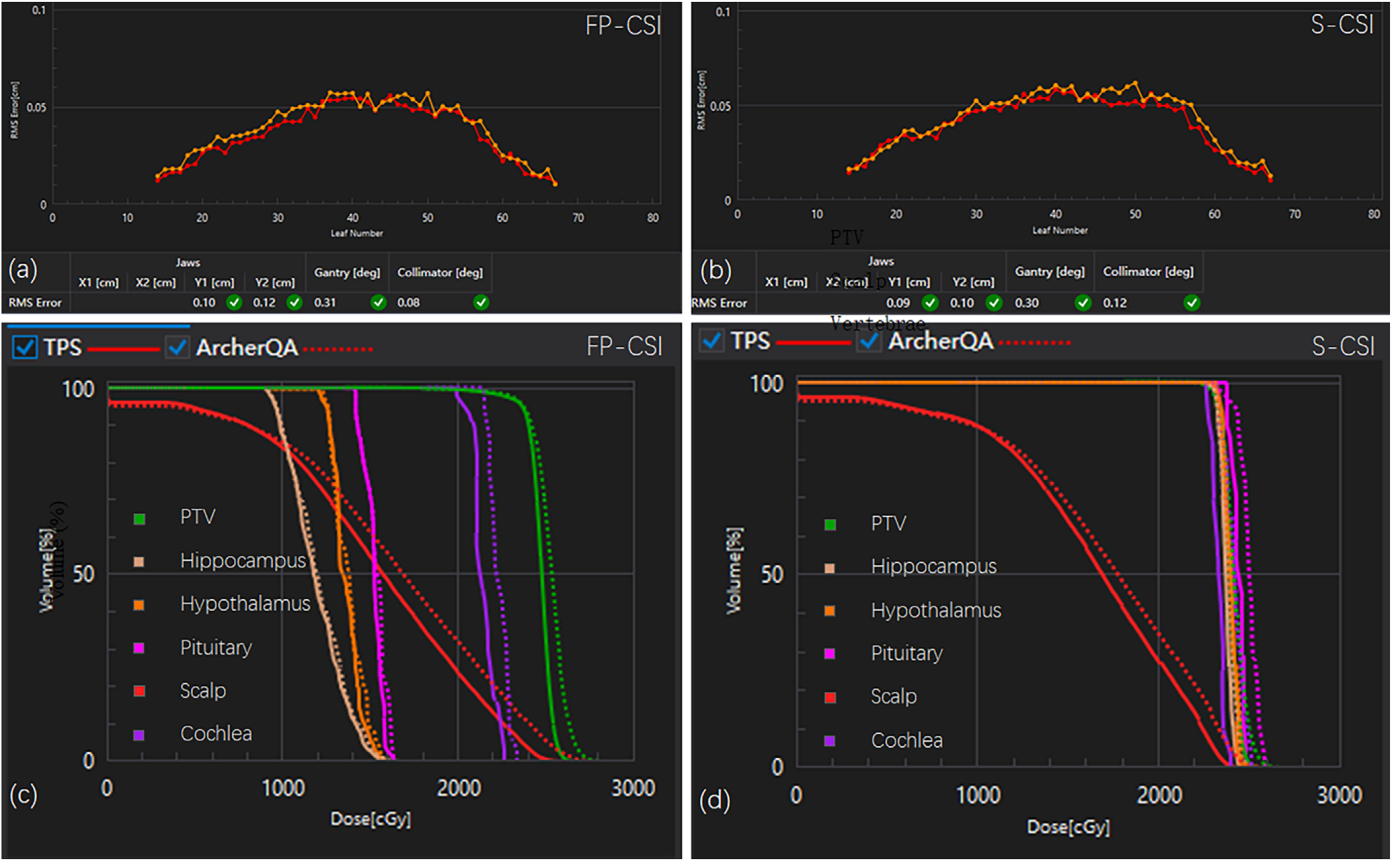
Log file–based analysis of mechanical accuracy (MLC, gantry, jaw, and collimator RMS errors) (a, b) and DVH comparison (c, d) between planned and reconstructed doses for FP‐CSI and S‐CSI plans in a representative patient. TPS: planned doses; ArcherQA: reconstructed doses. Dose values in the DVH are displayed in cGy due to system‐setting; all reported dose metrics in the manuscript are expressed in Gy for consistency. Due to substantial overlap among the DVH curves in panel (d), detailed DVH comparisons for the PTV and individual organs at risk (OARs) in the S‐CSI plan are presented separately in Supplementary Figure S1.

The 3D dose reconstruction performed using ArcherQA demonstrated small volumetric dose deviations from the planned doses for all evaluated structures (Figure 7). The DVH comparisons between planned and reconstructed doses showed close agreement, with deviations remaining within clinically acceptable limits. The root mean square (RMS) errors of the MLC positions were less than 0.6 mm, and the RMS errors of the gantry angles were less than 0.4°, indicating accurate mechanical performance during beam delivery. Overall, the dose deviations (ΔD) were within clinically acceptable limits for both FP‐CSI and S‐CSI plans.

Detailed dose deviations are summarized in Table 7. For the PTV, the dose deviations (ΔD) were below 1 Gy for all evaluated metrics. FP‐CSI showed slightly higher ΔD_98%_ values (0.6 ± 0.1 Gy) compared with −0.2 ± 0.1 Gy for S‐CSI (*p* < 0.01), indicating sufficient target coverage of FP‐CSI under real delivery conditions. For OARs, the volumetric dose deviations were generally within 1 Gy, confirming good delivery reproducibility.

**TABLE 7:** Volumetric dose deviations (ΔD_x_) between delivered doses reconstructed from log files and planned doses for FP‐CSI and S‐CSI plans.

Structures	Dose deviations	FP‐CSI	S‐CSI	*p*‐value
PTV	ΔD_95%_ (Gy)	0.1 ± 0.1	−0.1 ± 0.1	<0.01
	ΔD_98%_ (Gy)	0.6 ± 0.1	−0.2 ± 0.1	<0.01
	ΔD_2%_ (Gy)	0.9 ± 0.1	0.8 ± 0.1	<0.01
	ΔD_mean_ (Gy)	0.4 ± 0.04	0.3 ± 0.1	<0.01
Hippocampus	ΔD_40%_ (Gy)	0.3 ± 0.2	0.2 ± 0.1	0.11
Hypothalamus	ΔD_50%_ (Gy)	0.3 ± 0.2	0.03 ± 0.2	<0.01
Pituitary	ΔD_50%_ (Gy)	0.1 ± 0.3	0.4 ± 0.2	0.06
Cochlea	ΔD_50%_ (Gy)	0.9 ± 0.2	0.8 ± 0.2	0.23
Scalp	ΔD_50%_ (Gy)	0.9 ± 0.2	0.8 ± 0.2	0.21

Abbreviations: FP‐CSI, functional preservation craniospinal irradiation; S‐CSI, standard craniospinal irradiation.

## 4 DISCUSSION

This study compared a newly developed FP‐CSI with standard CSI in pediatric patients with MB. FP‐CSI achieved substantial dose reductions to critical functional organs, particularly the hippocampus and HPA, while maintaining clinically acceptable target coverage. Biological modeling further demonstrated that FP‐CSI markedly lowered the predicted risks of neurocognitive impairment and endocrine dysfunction compared with S‐CSI. Although FP‐CSI technique exhibited slightly higher plan complexity and reduced robustness, its overall delivery accuracy remained within clinical tolerance, confirming the feasibility of this function‐sparing approach in pediatric CSI.

The overall 5‐year survival rate for patients with average‐risk MB exceeds 80% with the combined use of surgery, CSI, and chemotherapy.^8^ However, ensuring a good quality of life remains a significant challenge for long‐term survivors. Increasing attention has been given to radiotherapy‐induced side effects, including cognitive decline, endocrine dysfunction, scoliosis, hearing loss, and permanent alopecia. To address these concerns, researchers have explored reduced‐dose CSI and selective dose‐sparing CSI techniques, such as hippocampal and HPA avoidance.^8^, ^9^, ^10^, ^11^ However, reduced‐dose CSI has been shown to be inferior to standard‐dose CSI in terms of event‐free survival and overall survival.^8^ Additionally, previous studies have not fully addressed other critical side effects, such as scoliosis, hearing loss, and permanent alopecia. In this study, we investigated the feasibility of sparing the hippocampus, HPA, scalp, and cochlea while maintaining homogeneous vertebral dose distribution in CSI. Our goal was to preserve the efficacy of radiotherapy while reducing long‐term side effects, ultimately improving the quality of life for pediatric MB patients.

Accurate target volume delineation is crucial for precise radiotherapy. Evidence suggests that inadequate coverage of the cribriform plate, temporal lobes, and inferior aspect of the thecal sac increases the risk of recurrence following CSI in MB.^29^, ^30^ Furthermore, cranial nerve foramina and canals may receive insufficient dose coverage unless specifically included in the CTV. In this study, the CTV delineation was performed to encompass the entire CSF space, following the SIOPE contouring guidelines.^12^ Accurate delineation of the hippocampus, HPA, scalp, cochlea, and vertebrae is also essential for achieving FP‐CSI. The methods and guidelines for contouring these structures are described in this study.^13^, ^14^, ^15^, ^16^, ^17^

FP‐CSI demonstrated a significant reduction in dose to functional structures. A comparison of published CSI techniques and the present FP‐CSI approach is provided in Table 8. The mean dose to the hippocampus decreased from 23.9 Gy in S‐CSI to 12.4 Gy in FP‐CSI. Although this dose was higher than the 10.6 Gy and 8.9 Gy reported in proton therapy by Gram et al.^31^ and Edvardsson et al.^32^, it was lower than the 17.2 Gy observed in VMAT plans by Zheng et al.^10^. Moreover, the maximum dose to the hippocampus was 16.5 Gy, and the minimum dose was 9.3 Gy, both of which are within the criteria suggested by the RTOG 0933 trial.^13^

**TABLE 8:** Comparison of CSI techniques from published studies and the present FP‐CSI approach.

Study	Prescription (Gy/fx)	Technique	Hippocampus D_mean_ (Gy)	HPA D_mean_ (Gy)	Cochlea D_mean_ (Gy)	Scalp D_mean_ (Gy)	Vertebrae D_2%_‐D_98%_ (Gy)
Seravalli et al. 2018^34^	36/20	3D‐CRT	—	—	—	31.2	—
		IMRT	—	—	—	32.3	—
		VMAT	—	—	—	28.1	—
		Tomo	—	—	—	30.9	—
		PBS	—	—	—	27.8	—
Zheng et al. 2020^10^	23.4/13	VMAT	17.2	13.9	—	—	—
		HT	15.9	15	—	—	—
Aljabab et al. 2021^11^	23.4/13	IMPT	14.7	14.1	—	—	—
Gram et al. 2023^31^	23.4/13	IMPT	10.6	—	—	—	—
Edvardsson et al. 2024^32^	23.4/13	IMPT	8.9	—	—	—	—
Hoeben et al. 2019^16^	≤25Gy	—	—	—	—	—	Posterior­anterior gradient < 5 Gy
FP‐CSI (this study)	23.4/13	VMAT	12.4	15	21.4	15.3	4.7

Abbreviations: FP‐CSI, functional preservation craniospinal irradiation; S‐CSI, standard craniospinal irradiation.

The maximum safe dose to the HPA is still under investigation. Merchant et al.^18^ reported that a cumulative dose of 16.1 Gy to the hypothalamus corresponds to a 50% risk of growth hormone deficiency at 5 years. Accordingly, we aimed to limit the mean dose to the hypothalamus and pituitary gland in FP‐CSI to below 16 Gy. The final plan achieved mean doses of 14.67 Gy and 15.36 Gy, respectively, values comparable to the 13.9–15 Gy range reported in VMAT and HT cohorts by Zheng et al.^10^ (Table 8).

Dose inhomogeneity in vertebral irradiation can result in long‐term spinal complications, including kyphosis, lordosis, scoliosis, and hypoplasia.^16^ In proton CSI, the entire vertebral body is included in the CTV to minimize sharp dose gradients for patients younger than 15 years.^33^ The dose homogeneity of vertebrae in CSI with photon has often been overlooked. However, Paulino et al.^4^ reported a 15‐year cumulative scoliosis incidence of 34.6% in children treated with photon CSI. In 2019, the SIOPE‐ROWG published consensus guidelines on vertebral delineation and dose constraints for pediatric patients, recommending posterior‐anterior dose gradients of less than 5 Gy for prescriptions of 25 Gy or less.^16^ In this study, the vertebral dose difference (D_2%_‐D_98%_) was limited to less than 5 Gy in FP‐CSI.

Hearing preservation following radiotherapy is closely related to the cochlear dose. The cochlear dose constraint was set at 21 Gy to prevent hot spots and ensure adequate coverage of the internal auditory canal, which lies adjacent to the cochlea and is included in the CTV. Permanent alopecia has also been associated with CSI dose. Torizuka et al.^6^ estimated the EQD 50% cutoff for severe permanent alopecia in pediatric MB patients to be 19.9 Gy. In FP‐CSI, the scalp D_50%_ was reduced to 15.27 Gy, compared to 16.4 Gy in S‐CSI, facilitating tumor bed boost optimization. This level is also lower than the scalp doses reported by Seravalli et al.^34^ (Table 8).

The selective sparing of functional OARs introduces risks of reduced homogeneity and conformity, including localized cold and hot spots. To control these effects and maintain acceptable conformity, ring structures were incorporated to shape dose fall‐off around the PTV and to limit unintended dose spill near avoidance regions. In parallel, explicit constraints on maximum dose and D_98%_ of PTV were applied during optimization, and iterative re‐optimization was performed whenever focal underdosage approached clinically relevant thresholds. In the final FP‐CSI plans, the D_2%_ of PTV was 25.5 Gy (below 110 % of the prescribed dose) and the near‐minimum dose (D_98%_) was 21.7 Gy (approximately 93% of the prescribed dose). These values fall within clinically acceptable limits for CSI and are unlikely to compromise tumor‐control probability despite the additional modulation required for functional avoidance.

In FP‐CSI, the cranial PTV D_98%_ was modestly reduced to 21.2 Gy; however, D_95%_ remained at 23.1 Gy, indicating that the decrease was limited to small regions adjacent to functional‐avoidance structures rather than reflecting global target under coverage. According to current pediatric CSI planning practice, target coverage is typically defined as D_95%_ ≥ 95% of the prescribed dose. Although D_98%_ is recommended as a metric to describe the near‐minimum dose, it is not commonly applied as a strict pass–fail criterion for plan acceptability.^31^, ^34^, ^35^ From a biological standpoint, the equivalent uniform dose (EUD) of the PTV was nearly identical between FP‐CSI and S‐CSI (23.6 Gy), indicating that overall tumor control probability (TCP) was preserved despite minor differences in near‐minimum dose (EUD and TCP calculation details are provided in the supplementary material Equations S1–S15 and Table S1). Therefore, this level of underdosage is considered clinically acceptable. Sparing of the cochlea and scalp had minimal impact on PTV coverage, not only due to their anatomical positions relative to the target volume but also because their dose constraints were relatively permissive (19 Gy of D_50%_ for the scalp and 21 Gy of D_mean_ for the cochlea).

The NTCP modeling results provide further evidence for the clinical advantages of FP‐CSI. The substantial reductions in hippocampal and hypothalamic–pituitary doses translated into markedly lower predicted risks of neurocognitive impairment and endocrine dysfunction, two of the most concerning late effects in pediatric MB survivors. Although NTCP models are inherently subject to interpatient variability and model uncertainties, they provide valuable biological insight supporting the clinical relevance of FP‐CSI dose reductions.

The robustness analysis demonstrated that FP‐CSI was more susceptible to setup uncertainties than S‐CSI, particularly under rotational perturbations. The tighter dose gradients around functional avoidance regions and the higher modulation complexity of FP‐CSI amplified the dosimetric impact of small angular deviations, leading to more pronounced variations in PTV and OAR doses compared with translational shifts. This finding underscores the need for precise patient immobilization and rigorous image‐guided verification, especially for maintaining hippocampal and hypothalamic–pituitary sparing. Incorporating robust optimization strategies or adaptive replanning could further mitigate the sensitivity to rotational errors while preserving the functional‐sparing benefits.

In this study, FP‐CSI plans exhibited greater modulation complexity than S‐CSI, particularly in the cranial fields. Higher MU, CPs, PI, and SAS10 values, combined with lower MCS, MFA, and MLG, indicated that FP‐CSI required finer beam modulation and smaller apertures to achieve improved dose conformity. Such characteristics are common in highly conformal IMRT/VMAT plans, where increased modulation enhances target shaping at the expense of greater delivery sensitivity. The regional analysis further showed that the cranial fields mainly contributed to this complexity, consistent with their more intricate anatomical geometry and proximity to critical organs.

The slightly lower gamma passing rates observed in FP‐CSI for cranial beams were consistent with its higher modulation complexity, suggesting a predictable relationship between plan complexity and deliverability. Nevertheless, all QA results and log file–based 3D dose reconstructions confirmed that both FP‐CSI and S‐CSI maintained delivery accuracy, with all deviations within clinically acceptable limits. These findings indicate that although FP‐CSI introduces more intricate modulation, its complexity remains well‐controlled and does not compromise treatment accuracy when supported by comprehensive QA procedures.

Although no validated complexity thresholds exist specifically for pediatric or adult CSI, published ranges from VMAT deliverability studies provide a useful reference for interpreting the present findings. Reported thresholds vary across TPS platforms and linac types, with PI commonly cited in the range of 9.8–11.1, MCS in 0.14–0.32, MLG in 1.9–2.5 cm, and SAS10 in 0.20–0.22.^25^, ^26^, ^36^, ^37^ In comparison, the cranial FP‐CSI beams in our study exhibited PI, MCS, MLG, and SAS10 values that remained within or close to these reported ranges, despite being more modulated than S‐CSI. Taken together, these comparisons suggest that while FP‐CSI increases cranial modulation, the overall plan complexity remains within expected clinical limits and is unlikely to compromise delivery reliability.

A key concern for FP‐CSI is the potential risk of disease recurrence in spared regions. Baliga et al.^38^ retrospectively analyzed recurrence patterns in 179 children with MB and reported no hippocampal failures, with only two recurrences occurring within 5 mm of the hippocampus. Similarly, Lucus et al.^39^ reported progression patterns in 155 MB patients, noting that metastatic failure typically occurred in the cerebellum, brainstem, and fourth ventricle, with infrequent involvement of the hypothalamus and pituitary gland for average‐risk patients.

The cochlea and scalp, located outside the CSF space, were additionally spared in FP‐CSI without increasing the risk of relapse. Although improving vertebral dose homogeneity slightly increased the dose to other thoracic and abdominal OARs, the difference was clinically acceptable (less than 1 Gy). Importantly, these adjustments did not compromise PTV coverage and are unlikely to increase the potential risk of normal tissue toxicity.

This study has several limitations. First, the sample size was small. Second, the boost phase to the tumor bed was not included, as the aim was to isolate the dosimetric effects of functional sparing within the CSI portion; incorporating boost fields would introduce case‐specific variability that could obscure these comparisons. Third, FP‐CSI is designed for average‐risk pediatric MB patients and may not be suitable for high‐risk groups or other conditions requiring CSI, such as germ cell tumors. Fourth, several NTCP models used in this study were originally developed from adult or non‐CSI cohorts, and their application to pediatric CSI introduces inherent uncertainties. Therefore, the NTCP values should be interpreted as comparative rather than absolute predictions. Finally, this work represents a treatment‐planning study, and the clinical significance of the observed dose differences—including both OAR sparing and minor reductions in PTV coverage—has not yet been evaluated. Prospective clinical validation will be required.

## 5 CONCLUSIONS

This study introduces a new radiation therapy technique, FP‐CSI, which incorporates “staggered overlap” and multi‐objective hierarchical optimization. This approach effectively reduces radiation doses to critical structures such as the hippocampus, HPA, cochlea, and scalp, while enhancing dose uniformity across the vertebrae. Importantly, it achieves these benefits while maintaining clinically acceptable target coverage in pediatric patients with average‐risk MB. As a result, FP‐CSI holds the potential to mitigate late radiotherapy‐induced side effects and significantly improve patients' quality of life.

## AUTHOR CONTRIBUTIONS

*Conception and design of the study*: Keqiang Wang, Wenxue Zhang; *Acquisition of data*: Keqiang Wang, Jie Chen, Jianbo Jian, Peng Wang, Hongyang Zhang; *Analysis of data*: Jie Chen; *Wrote the paper*: Keqiang Wang; *Critical review*: Wenxue Zhang; Final approval of the manuscript: All authors.

## CONFLICT OF INTEREST STATEMENT

The authors have no relevant conflicts of interest to disclose.

## ETHICS STATEMENT

This retrospective study was reviewed and approved by the Institutional Review Boards of Tianjin Medical University General Hospital (IRB2024‐YX‐137‐01) and the requirement for individual informed consent was waived.

## Supporting information



Supporting Information
