# Supplementary material for: A feasibility study of functional preservation in craniospinal irradiation with photon for pediatric medulloblastoma
Source: J Appl Clin Med Phys. 2026 Jan 27;27(2):e70474. doi: 10.1002/acm2.70474 (PMC12836293; doi:10.1002/acm2.70474)
Supplement: Supplementary file 1 — Supporting Information [file ACM2-27-e70474-s001.docx]

**Supplementary material**

1. **Plan-averaged beam irregularity (PI)**

Plan-averaged beam irregularity (PI) quantifies deviations of aperture shapes from a perfect circle; PI = 1 corresponds to a circular aperture. For beam b and segment s the aperture irregularity is defined as:

$$\begin{aligned} {AI}_{bs}=\frac{{AP}_{bs}^{2}}{4\pi\cdot{AA}_{bs}}\#\left( 1 \right) \end{aligned}$$

where AP_bs_ is the aperture perimeter and AA_bs_ is the aperture area. The beam irregularity for beam b is the MU-weighted average over its S segments:

$$\begin{aligned} {BI}_{b}=\frac{\sum_{s=1}^{S} {MU}_{bs}{AI}_{bs}}{{MU}_{b}}\#\left( 2 \right) \end{aligned}$$

The plan irregularity is then the MU-weighted average across all B beam: $\begin{aligned} PI=\frac{\sum_{b=1}^{B} {MU}_{b}{BI}_{b}}{{MU}_{plan}}\#\left( 3 \right) \end{aligned}$

1. **Modulation complexity score (MCS)**

We used the modulation complexity score (MCS) as originally proposed by McNiven et al. and later adapted for VMAT by Masi et al. The MCS reflects both aperture area variability (AAV) and leaf sequence variability (LSV). For a control point (or segment) cp, the aperture area variability (AAV) is defined as:

$$\begin{aligned} {AAV}_{cp}=\left( \frac{\sum_{1}^{N} \left( p_{i,left_{bank}}-p_{i,right_{bank}} \right)}{\sum_{1}^{N} ({max(p}_{i,left_{bank}})-{max(p}_{i,right_{bank}}))} \right)\#\left( 4 \right) \end{aligned}$$

where p_i,leftbank_ and p_i,rightbank_ are the coordinates (or positions) of the left and right leaf of leaf pair i at that control point, N is the number of leaf pairs.

Leaf sequence variability (LSV) for a control point represents the variability in the area of segments:

$$\begin{aligned} {LSV}_{cp}=\left( \frac{\sum_{1}^{N-1} \left( p_{max}-\left| {(p}_{i}-p_{i+1}) \right| \right)}{\left( N-1 \right)\times p_{max}} \right)_{left_{bank}}\times\left( \frac{\sum_{1}^{N-1} \left( p_{max}-\left| {(p}_{i}-p_{i+1}) \right| \right)}{\left( N-1 \right)\times p_{max}} \right)_{right_{bank}}\#\left( 5 \right) \end{aligned}$$

where p_i_ is the coordinate of the ith leaf position, p_max_ is the maximum distance between positions for a given leaf bank, summed over all control point.

The MCS for an arc is then the product of LSV and AAV weighted by the relative number of monitor units:

$$\begin{aligned} {MCS}_{arc}=\sum_{1}^{N} \left[ \left( \frac{{AAV}_{cp,i}+{AAV}_{cp,i+1}}{2} \right) \right]\times\left( \frac{{LSV}_{cp,i}+{LSV}_{cp,i+1}}{2} \right)\times\frac{{MU}_{cpi,i+1}}{{MU}_{arc}}\#\left( 6 \right) \end{aligned}$$

where MU_cpi,i+1_ indicates the MUs delivered between two successive control points (cpi and cp(i+1)).

MCS ranges from 0 to 1, with values near 1 indicating simple (unmodulated) fields and values near 0 indicating highly modulated fields.

1. **Small aperture score (10 mm)-** **SAS10**

The small aperture score SAS10 is the MU-weighted fraction of leaf pairs with an aperture less than 10 mm among all open leaf pairs. For a beam:

$$\begin{aligned} {SAS10}_{beam}=\sum_{i=1}^{I} \frac{N\left( 10>a>0 \right)_{i}}{N\left( a>0 \right)_{i}}\times\frac{{MU}_{i}}{{MU}_{beam}}\#\left( 7 \right) \end{aligned}$$

where I is the number of segments in the beam, N is the number of leaf pairs not positioned under the jaws, and a is the aperture distance between opposing leaves.

1. **Mean field area (MFA)**

The mean field area (MFA) for a beam is the MU-weighted mean of the aperture areas across all segments:

$$\begin{aligned} {MFA}_{beam}=\sum_{i=1}^{I} A_{i}\times\frac{{MU}_{i}}{{MU}_{beam}}\#\left( 8 \right) \end{aligned}$$

where I is the number of segments in the beam and A_i_ the aperture area of segment i.

1. **Mean leaf gap (MLG)**

Mean leaf gap (MLG) quantifies the average distance between opposing (in-field) leaf pairs, MU-weighted across all control points. A clear expression is:

$$\begin{aligned} MLG=\frac{\sum_{k=1}^{N_{cp}} \sum_{l=1}^{N_{leaf}} \left| p_{l,right_{bank}}-p_{l,left_{bank}} \right|_{k}\cdot{MU}_{k}}{\sum_{k=1}^{N_{cp}} \sum_{l=1}^{N_{leaf}} {MU}_{k}}\#\left( 9 \right) \end{aligned}$$

where N_cp_ is the number of control points, N_leaf_ is the number of leaf pairs.

1. **Mean leaf travel (MLT)**

Mean leaf travel measures the average leaf motion over the arc. First, for a transition between s-1 and s (LTs) is calculated with equation:

$$\begin{aligned} {LT}_{s}=\frac{\sum_{l=1}^{N_{leaf}} \left| X_{l,s}-X_{l,s-1} \right|}{N_{s}}\#\left( 10 \right) \end{aligned}$$

where x_s_ and x_s-1_ are the MLC positions for segment s and segment s-1 respectively, N_s_ is the number of MLC leaves in the segment and l is the leaf index.

Subsequently, LT for a beam is calculated with equation:

$$\begin{aligned} {LT}_{b}=\frac{\sum_{s} {LT}_{s}}{N_{b}}\#\left( 11 \right) \end{aligned}$$

where N_b_ is the number of segments in the beams and s is the segment index.

Finally, LT for a plan is calculated with equation:

$$\begin{aligned} MLT=\frac{\sum_{b} {LT}_{b}}{N_{p}}\#\left( 12 \right) \end{aligned}$$

where N_p_ is the number of beams in the plan and b is the beam index.

**Equivalent uniform dose (EUD) and tumor control probability (TCP)**

The EUD was calculated according to Equations (13) and (14):

$$\begin{aligned} EUD=\left( \sum_{i} v_{i}{EQD}_{2i}^{a} \right)^{\frac{1}{a}}\#\left( 13 \right) \end{aligned}$$

$$\begin{aligned} {EQD}_{2i}=d_{i}\cdot\frac{\frac{d_{i}}{n_{f}}+\frac{\alpha}{\beta}}{2+\frac{\alpha}{\beta}}\#\left( 14 \right) \end{aligned}$$

where $v_{i}$ is the relative subvolume receiving the equivalent dose ${EQD}_{2i}$. The ${EQD}_{2}$ represents the biologically equivalent dose in 2Gy fractions and depends on the dose per voxel $d_{i}$, number of fractions $n_{f}$ and the $\alpha/\beta$ ratio. In this study, the parameters were set as a= -10, $n_{f}$= 13 and $\alpha/\beta$= 10Gy.

The TCP was calculated using the following expression:

$$\begin{aligned} TCP=\prod_{j=1}^{R} \left\{ \frac{1-P_{0,j}}{1+\left( \frac{D_{50,j}}{D_{j}} \right)^{{4\gamma}_{50,j}}}+P_{0,j} \right\}\#\left( 15 \right) \end{aligned}$$

with parameter values derived from Gram et al. 2023 (31):

$\gamma_{50,elective}=0.36$,

$\gamma_{50,boost}=0.36$,

$P_{0,elective}=0.695$,

$P_{0,boost}=0.716$,

$D_{50,elective}=17Gy$,

$D_{50,boost}=40.36Gy$,

and $D_{boost}$=54Gy.

**Table S1** Comparison of predicted TCP values and corresponding EUD between FP-CSI and S-CSI

| Parameters | FP-CSI | S- CSI | *p*-value |
| --- | --- | --- | --- |
| EUD (Gy) | 23.6 ± 0.2 | 23.6 ± 0.1 | 0.27 |
| TCP (%) | 78.4 ± 0.1 | 78.3 ± 0.02 | 0.27 |

TCP, tumor control probability; EUD, equivalent uniform dose; FP-CSI, functional preservation craniospinal irradiation; S-CSI, standard craniospinal irradiation


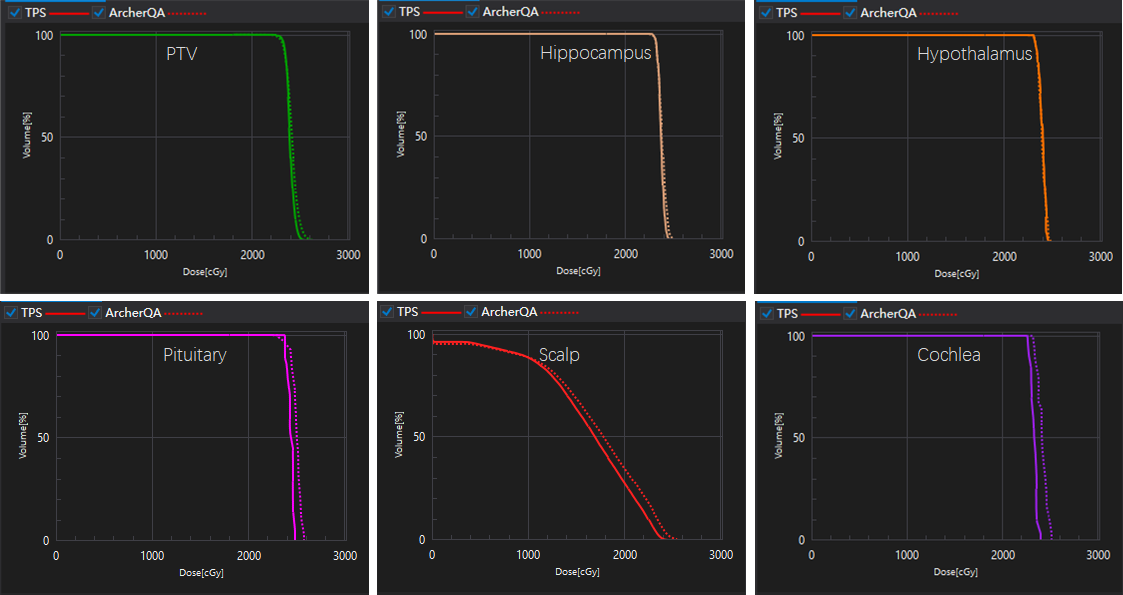


**Fig. S1.** DVH comparisons for the PTV and individual organs at risk (OARs) between log file–based reconstructed doses (ArcherQA) and planned doses (TPS) in the S-CSI plan of a representative patient.
